# Management of iatrogenic chloroform mucosal burn in the palate: a case report

**DOI:** 10.34172/japid.2022.004

**Published:** 2022-04-10

**Authors:** Ali Taghavi Zenouz, Katayoun Katebi, Mohammad Ali Ghavimi, Farshad Javadzadeh, Maryam Hosseinpour Sarmadi

**Affiliations:** ^1^Department of Oral Medicine, Faculty of Dentistry, Tabriz University of Medical Sciences, Tabriz, Iran; ^2^Department of Oral and Maxillofacial Surgery, Faculty of Dentistry, Tabriz University of Medical Sciences, Tabriz, Iran

**Keywords:** Chloroform, iatrogenic disease, chemical burn, case report

## Abstract

Chloroform is used widely in endodontic treatments for solving gutta-percha points, but it can have destructive effects if it comes in to contact with oral mucosa. This article presents a case of necrotic ulcer of palatal and buccal mucosa due to injudicious use of chloroform in endodontic treatment, which has caused severe destruction in maxilla. A conservative treatment method of repeated curettage and irrigation was used and although the lesion healed completely, it had major effects on the patient’s quality of life including loss of two teeth. It is important that dentist be aware of the devastating effects of imprudent application of various chemicals used in dentistry. The conservative treatment used for this case can be helpful option for similar cases.

## Introduction

 Injuries to oral mucosa can occur due to accidental, Iatrogenic and factitious trauma. The injuries may present as burns, ulcerations and gingival recessions.^1^ The main causative agents for oral mucosal burns are the chemical and thermal ones. Various chemical materials are used in dentistry and many of them can harm the oral mucosa, if used injudiciously.^2^

 Chemical injuries can have different clinical presentations based on the composition and concentration and PH of the chemical agent. Also the quantity of the used substance and duration of tissue contact have an important role on the extent of injury^2^ The mucosal lesions can range from a local erosion to complete mucosal detachment and they can extend to submucosa leading to permanent scarring.^3^

 There are some case reports in medical literature regarding chemical injuries in oral mucosa including paraformaldehyde, formocresol and sodium hypochlorite. We found three articles about chloroform iatrogenic burns related to dentistry, although their presentations, management and outcomes were different from the present case.^4-6^This paper presents a case of an ulcer in palatal and buccal mucosa due to chloroform injection in to the root canal in endodontic treatment and discusses its conservative treatment and effects on the patient’s quality of life.

 This case report follows the consensus-based clinical case reporting guideline (CARE) checklist (https://www.care-statement.org/).^7^

## Case report

**Figure 1:**
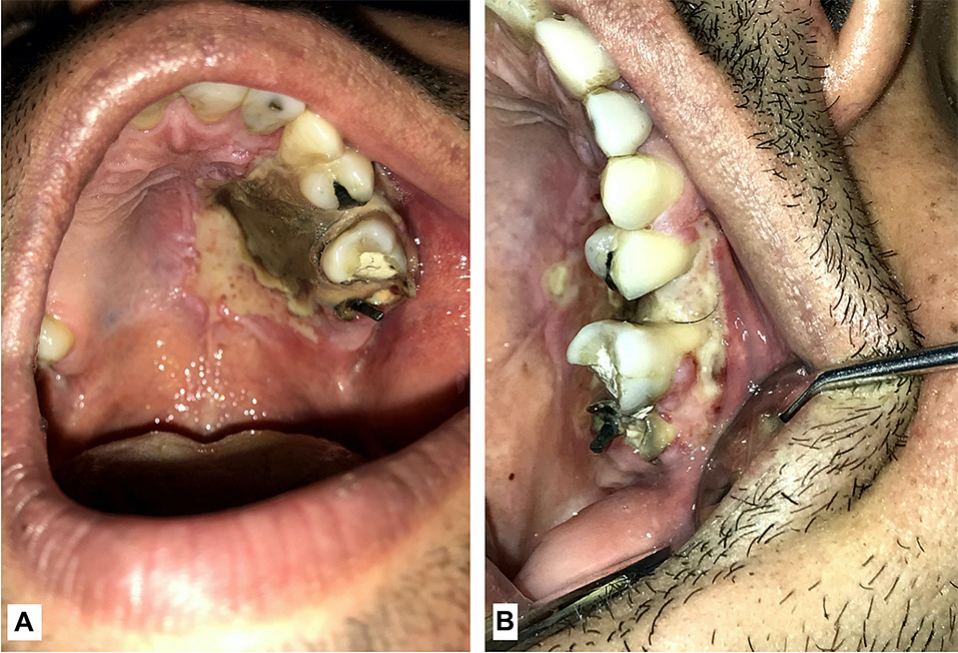
Ulceration of the palatal mucosa (A) and buccal mucosa (B) due to chloroform (first visit).

 A 30 years old male presented to the oral medicine department of Tabriz University of medical sciences with an extensive ulcer in the left half of the palatal mucosa (Figure 1-A) and a second ulcer on the buccal vestibule of maxillary left first molar (Figure 1-B).

 He was an athlete in good general health and his medical history revealed no previous medical condition. He had received a root canal treatment of maxillary left first molar three days earlier and had a severe pain during these three days. For finding the causing agent the dentist was contacted, it was revealed that he had injected chloroform in to the root canal of maxillary left first molar for anesthesia whitout using a proper isolation method like rubber dam.

 The clinical examination revealed a necrotic ulcer on the hard palate extending from the mesial aspect of the maxillary left canine tooth to mesial aspect of maxillary second molar. On the buccal region the ulcer was extended from mesial aspect of maxillary left first premolar to mesial aspect of maxillary second molar exposing the root of the maxillary first molar. The palatal ulcer was very painful and the patient has not been able to eat solid food in three days.

 In a consultation with an oral and maxillofacial surgen, it was suggested that due to the extension of the lesion a hemi-maxillectomy may be needed if it did not respond to curettage.

**Figure 2:**
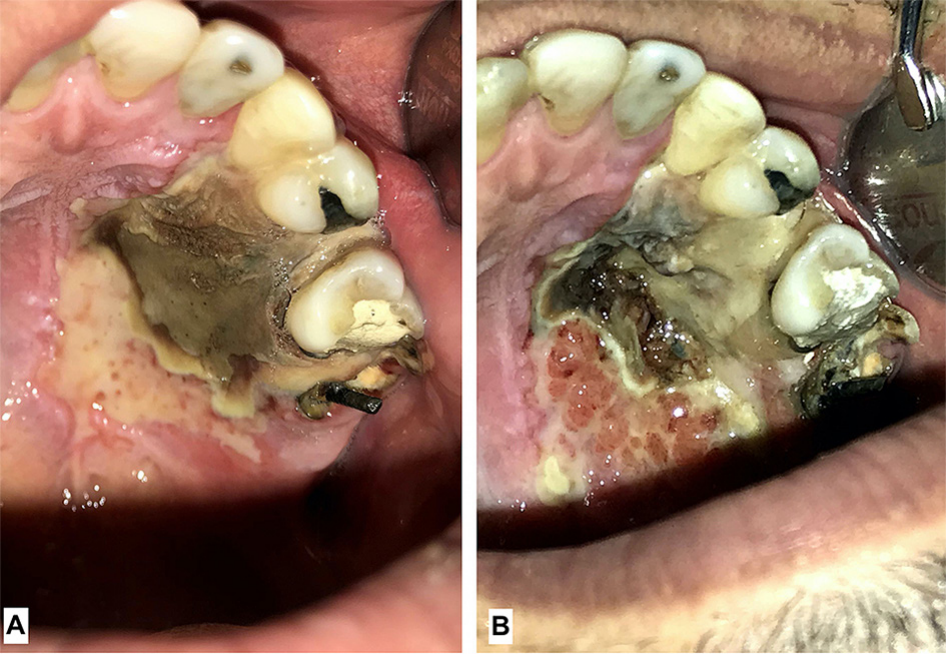
Palatal mucosa in second (A) and third visit(B).

**Figure 3:**
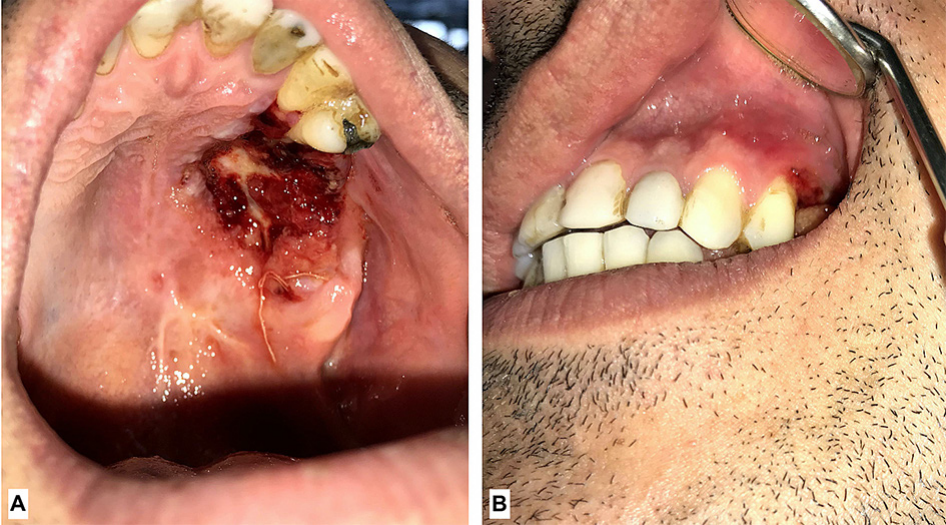
Palatal mucosa (A) and buccal mucosa (B) in fifth visit.

**Figure 4:**
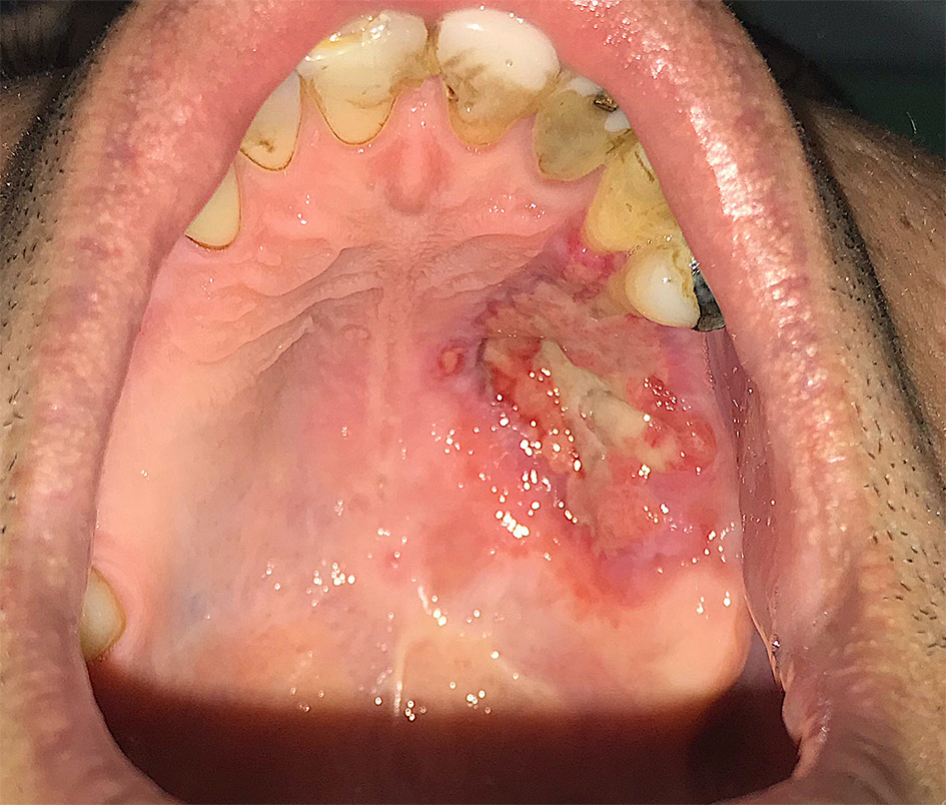
Healing after 1 month in sixth visit.

 The palatal ulcer was curetted until bleeding started and the patient was prescribed a sterile saline for irrigation and 500 mg Amoxicillin capsules every 8 hours and 400 mg Ibuprofen tablets every 6 hours. Also, meticulous oral hygiene including frequent use of sterile saline irrigation and teeth brushing to the point that was tolerable was advised. After 48 hours patient was visited. The palatal ulcer has shrunk (Figure 2A) and the pain level has increased. A second curettage was performed and 500 mg Acetaminophen tablets every 6 hours was added to previous regimen. The patient was followed up 72 hours later and it was evident that the ulcer was shrinking (Figure 2B). In 4^th^ session the maxillary first and second molars were extracted and more debridement was conducted on necrotic tissue. In the 5^th^ follow up session ( day 14^th^since first visit) patient reported that his pain was relieved and the size of the lesion had considerably decreased. (Figures 3). In the 6th follow up session (day 30th since first visit) The lesion was healing ( Figure 4). It took about two months for complete healing of the area. Due to traveling problems, the patient didn’t return for further follow ups and reported the results of healing by phone. It took about two months for complete healing of the area.

 Written informed consent was obtained from patient to report this case.

## Discussion

 Chemical injuries of the oral soft tissues may be caused by different substances. The extent of lesions caused by chemical agents depends on the type, contact time, concentration and quantity of the causing agent.^8^ In dentistry there are various substances, such as hydrogen peroxide, sodium hypochlorite, calcium hydroxide and formocresol solutions which can cause injuries if used negligently.^9^

 Chloroform is used in endodontics for solving gutta-percha points and for removal of gutta-percha from root canals in re-treatments,^10^ but in this case it was used as anesthetic agent by being injected into root canal of the maxillary first molar. In a case report by Akhlaghi et al the chloroform was used in retreatment but there was a perforation in the root which caused chloroform leakage.^4^ Mubarak Alkahtany^5^ reported a case of skin burn due to chloroform. Both of these cases were retreatments unlike the present case.

 Verma et al reported a case in which chloroform was injected accidentally instead of local anesthesia because chloroform was loaded into the anesthetic syringe.^6^ But in the present case the dentist had used chloroform in an attempt to reach additional pulpal anesthesia.

 Many substances are used in dentistry, therefore cautious handling of the materials is very crucial, furthermore, proper isolation techniques such as the use of rubber dam can provide a protection for oral tissue.

 Treatment of oral chemically induced ulcerations requires identification of the causative agent and its removal if possible.^11,12^ Plaque accumulation over the injured area should be prevented by careful oral hygiene measures and frequent irrigation;^13^ and topical anesthetic agents can improve the symptoms.^14^ Prophylactic antibiotic prescription is also recommended.^15^ The most commonly used irrigation agent is chlorhexidine^11,13^but this patient could not tolerate the chlorhexidine. Therefore saline was prescribed instead. In hard tissue damages, the necrotic area should be removed surgically in order to accelerate repair and subsequent regeneration.^16^ These patients should be encouraged to consume plenty of fluids and soft diet and a rich diet. These measures are necessary for maintaining the general wellness of the individual as in this case patient lost 5 kg weight in the first two weeks due to inability to consume solid food.

## Conclusion

 Dentists should be aware of the effects of unconsidered application of dental materials. Permeant damages of chemical burns can be prevented if isolation techniques such as rubber dam are used and the exposure is detected early and management requires identification of causing agent and its removal and perfect oral hygiene measures.

## Patient’s perspective

 I am an athlete and I do bodybuilding. In the dental office while receiving endodontic treatment I sensed an odd smell in an injection, which was not home bleaching material’s smell. Three days later there was a large and painful ulcer in my palate. After the first therapy session in Tabriz faculty of dentistry, my pain level increased significantly, but the doctors said it was a good sign and meant the nerve injury was not severe. It took a long time for my mouth to heal completely and in those times, I lost a lot of weight and I couldn’t exercise. I hope this kind of accident doesn’t happen to anyone

## Authors’ contributions

 AT supervised the treatments and treatment plan, KK conducted the literature search and pharmacological treatments, MG carried out the surgical treatments, FJ took and prepared the clinical photographs and contributed to manuscript preaparation and MH prepared the initial draft of the manuscript. All authors have read and approved the final manuscript.

## Funding

 Not applicable.

## Availability of data

 The anonymous data of the present case is available from the corresponding author on request.

## Consent for publication

 The patient gave a signed informed consent for the treatments and publishing of this report.

## Competing interests

 The authors declare that they have no competing interests.
